# Correlates of Fitness Tracker Ownership and Use in Cancer Survivors: Cross-Sectional Survey

**DOI:** 10.2196/92876

**Published:** 2026-05-26

**Authors:** Roberto M Benzo, James L Fisher, Macy K Tetrick, Rujul Singh, Alex B Osei, Jessica Krok-Schoen, Brett S Nickerson, Electra D Paskett, Ali Kargarandehkordi, Sachin Kumar, Sara M St George, Michael F Rayo, Frank J Penedo, Peter Washington

**Affiliations:** 1 Division of Cancer Prevention and Control, Department of Internal Medicine College of Medicine The Ohio State University Columbus, OH United States; 2 The Ohio State University Comprehensive Cancer Center The Ohio State University Wexner Medical Center Columbus, OH United States; 3 Arthur G. James Cancer Hospital Columbus, OH United States; 4 Grossman School of Medicine New York University New York, NY United States; 5 Department of Design The Ohio State University Columbus, OH United States; 6 School of Health and Rehabilitation Sciences The Ohio State University Columbus, OH United States; 7 Department of Information and Computer Sciences University of Hawaiʻi at Mānoa Honolulu, HI United States; 8 Division of Clinical Informatics and Digital Transformation (DoC-IT) Department of Medicine University of California San Francisco, CA United States; 9 Department of Integrated Systems Engineering College of Engineering The Ohio State University Columbus, OH United States; 10 Department of Public Health Sciences Miller School of Medicine University of Miami Miami, FL United States; 11 Sylvester Comprehensive Cancer Center University of Miami Miller School of Medicine Miami, FL United States; 12 Department of Computer Sciences and Engineering College of Engineering The Ohio State University Columbus, OH United States; 13 Department of Psychology University of Miami Miami, FL United States

**Keywords:** digital health, cancer survivorship, wearable devices, physical activity, behavior change, usability, mobile health, mHealth, fitness tracker, wearable activity monitors, health equity, US adult population, sociodemographic determinants of physical activity, wearables, United States, observational studies, sociodemographic determinants, physical activity data

## Abstract

**Background:**

Consumer fitness tracker devices offer scalable opportunities to monitor real-world behavior and support health in cancer survivorship. However, adoption and sustained use outside structured research settings remain incompletely characterized, limiting their integration into survivorship care.

**Objective:**

The primary objectives were to describe the prevalence of fitness tracker ownership and use patterns among cancer survivors and to identify sociodemographic, psychosocial, and usability-related correlates of device ownership and frequent use.

**Methods:**

We conducted a cross-sectional survey of 893 cancer survivors enrolled in the Total Cancer Care protocol at a comprehensive cancer center. Participants completed an adapted online questionnaire assessing fitness tracker ownership, frequency of use, and perceived barriers and facilitators. Multivariable logistic regression models were used to identify sociodemographic, psychosocial, and usability-related correlates of device ownership and frequent use.

**Results:**

More than half of the participants (506/893, 56.7%) reported owning a fitness tracker, and among owners, 82.2% (416/506) reported frequent use (most days or every day), including 71.3% (361/506) who wore the device every day. The most commonly used devices were the Apple iWatch (272/506, 53.8%) and Fitbit (147/506, 29.1%). In multivariable analyses, fitness tracker ownership was independently associated with sex, household income, and cancer site. Male participants had lower odds of ownership (adjusted odds ratio [aOR] 0.61, 95% CI 0.42-0.87; *P*=.006), while higher household income was associated with greater ownership (US $50,000-$99,999: aOR 1.70, 95% CI 1.13-2.57; *P*=.01; ≥US $100,000: aOR 3.64, 95% CI 2.41-5.50; *P*<.001). Ownership also differed by cancer site. Among owners, frequent use was concurrently and inversely associated with self-reported device discomfort (*P*<.001), low motivation (*P*<.001), information overload (*P*=.04), and limited app integration (*P*=.007).

**Conclusions:**

In this single-center, predominantly White, higher-income sample of cancer survivors, fitness tracker ownership was common and patterned by demographic and socioeconomic characteristics, while sustained engagement among device owners was associated with psychosocial and usability factors. These findings suggest that scalable, fitness tracker–enabled survivorship care will need to address both structural disparities in ownership as well as behavioral readiness and user experience to ensure clinically meaningful implementation.

## Introduction

Fitness tracker sensing technologies have become increasingly prominent in oncology research as tools for continuously capturing real-world physiological and behavioral data, including physical activity (PA), heart rate, heart rate variability, sleep, and circadian rest-activity rhythms. These devices offer a potentially scalable approach for monitoring patient recovery, functional status, and emerging health risks in daily life, complementing infrequent clinic-based assessments [1-3]. Their expanding role reflects a broader shift toward digital health measurement in cancer survivorship and the need for objective data streams that can inform timely intervention and long-term self-management.

PA is among the most frequently assessed metrics in fitness tracker–enabled oncology research because of its established relevance to cancer survivorship outcomes. Moreover, exercise is safe before, during, and after treatment and is associated with improved quality of life, reduced fatigue, and better physical function [3,4]. Observational studies consistently link higher postdiagnosis PA with lower risks of cancer-specific and all-cause mortality across multiple cancer types [3,5]. However, many survivors experience declines in PA during treatment that persist into survivorship, and exercise promotion is not routinely embedded in oncology care [6]. Reflecting this gap, Stout et al [7] highlighted the need for patient-centered pathways that support repeated assessment of functional status and timely referral for personalized exercise interventions. In this context, fitness tracker devices may complement clinic-based performance tests by enabling continuous monitoring of functional proxies, such as habitual and ecologically valid measures of PA walking patterns and heart-rate responses to daily ambulation, as well as emerging estimates of cardiorespiratory fitness (eg, maximum volume of oxygen consumed [VO₂max] derived from walking-based algorithms), thereby supporting scalable identification of functional decline between visits.

Recent reviews indicate substantial growth in the use of fitness trackers across oncology studies. In a synthesis of 199 trials involving more than 18,500 patients, Chow et al [8] reported that the most commonly used devices were ActiGraph (36% of studies), Fitbit (19% of studies), Garmin (7% of studies), and activPAL (6% of studies) [8]. Activity minutes and step counts were the most frequently collected metrics, with adherence rates reported at or above 80%, when available. Complementary work has emphasized the role of fitness trackers in behavioral interventions: a meta-analysis of randomized controlled trials found that programs incorporating pedometers or activity trackers yield significant increases in moderate-to-vigorous PA, total activity, and daily steps, as well as improvements in fatigue, aerobic fitness, and quality of life relative to usual care [9]. A scoping review concluded that wearable fitness trackers are generally acceptable and can facilitate short-term increases in activity in cancer survivors, although long-term adherence and maintenance remain inconsistent [10].

Tracker-derived signals may also provide insight into broader health outcomes beyond PA. For example, rest-activity rhythm parameters obtained from accelerometry have demonstrated moderate-to-strong associations with survival, functional status, and symptom burden [11]. However, the overall literature remains limited by methodological heterogeneity, reliance on small or trial-selected samples, variability in device algorithms, and minimal integration into routine survivorship care [8,10,11]. These limitations underscore the need to understand the real-world feasibility of relying on fitness tracker devices to inform clinical or behavioral decision-making.

A critical barrier to translation is that little is known about real-world patterns of fitness tracker ownership and use among cancer survivors. Most existing oncology studies recruit participants who agree to use devices provided by the research team, leaving unanswered questions on whether survivors in the general population own fitness trackers, use them consistently, or differ from the broader population in meaningful ways. Moreover, digital health interventions risk reinforcing inequities if fitness tracker access and engagement vary by age, race, ethnicity, socioeconomic status, or cancer history. Characterizing these patterns is essential for determining the feasibility, reach, and equity of tracker-enabled survivorship care.

To address these gaps, this study examined fitness tracker ownership and active use among a large sample of cancer survivors. We identified sociodemographic, clinical, and behavioral correlates of ownership and sustained use, providing data needed to inform the design and implementation of equitable, scalable digital health strategies for cancer survivorship.

## Methods

### Study Design and Setting

This cross-sectional study examined fitness tracker ownership and use among cancer survivors enrolled in the Total Cancer Care (TCC) protocol at the Ohio State University Comprehensive Cancer Center – James Cancer Hospital and Solove Research Institute. The study followed the CONSORT (Consolidated Standards of Reporting Trials) guidelines. Data were collected between December 2023 and December 2024 using a self-administered online survey delivered through REDCap (Research Electronic Data Capture). A trained research assistant was available over email to address participants’ questions.

### Ethical Considerations

The study was approved by the Ohio State University Institutional Review Board (IRB #2023C0190) with a waiver of signed consent, and standardized protection of participants’ rights and interests was ensured. Online consent was obtained through survey completion. The right to discontinue involvement in the survey at any point was guaranteed to participants. Participants were offered entry into a raffle for US $25 Amazon electronic gift cards as compensation for their participation. Data security and participant confidentiality were protected via several measures: data were fully deidentified, secure and password-protected data storage systems were installed, access to identifying information was implemented, and confidentiality protocols were mandated.

### Participants and Recruitment

Eligible participants were adults (≥18 years), English speaking, diagnosed with a neoplasm (breast, prostate, lung, colorectal, head and neck, gynecologic, genitourinary, endocrine, hematologic, thoracic, digestive, nervous system, cutaneous, or soft-tissue/bone sites), and with an active email address in the TCC registry. Individuals with cognitive or psychiatric impairments precluding informed participation were excluded.

Recruitment was conducted by querying the TCC database for all eligible patients who had not been recently contacted for other survey requests (ie, ≥3-4 months since the last contact). The sampling frame included 8568 patients with cancer at different sites: breast (n=2137, 24.9%), thyroid (n=1451, 16.9%), head and neck (n=1670, 19.5%), digestive system (n=661, 7.7%), urinary system (n=727, 8.5%), thorax (n=409, 4.8%), hematologic system (n=533, 6.2%), gynecologic system (n=190, 2.2%), and additional sites (cutaneous, nervous system, soft-tissue/bone, and unknown; n=790, 9.2%). Of these, 7700 (89.9%) individuals had an email address registered with the TCC and were contacted via email invitations sent in monthly batches. Participants received an email invitation with a short study description and a link to a landing webpage with a full description.

Of the 7700 (89.9%) individuals contacted, 895 (11.6%) completed the survey, 152 (2.0%) partially completed the survey, and 6653 (86.4%) did not respond. Two completed surveys were excluded due to missing key demographic variables required for analysis, yielding a final analytical sample of 893 (11.6%) participants. The overall survey response rate (completed or partially completed surveys) was 13.6% (n=1047).

### Survey Instrument

The survey was part of the Lifestyle Technology in Cancer Study, a broader multimodule questionnaire designed to assess how cancer survivors engage with mobile health (mHealth) technologies across several domains.

For the fitness tracker component, an instrument developed by Holko et al [12] for Federally Qualified Health Center patients was used and adapted with permission. The adaptation retained the original structure (awareness, ownership, reasons for use and nonuse, perceived barriers, and data-sharing preferences), while updating language to reflect survivorship contexts (eg, anchoring items to recovery and health after cancer treatment) and modern device features, such as sleep tracking, workout guidance, and integration with other apps and devices. Additional items were added to capture survivor-specific considerations, including participants’ interests in topics, format, and features for future digital lifestyle interventions. Prior engagement with supportive care services, including nutrition and PA counseling, was assessed in alignment with American Cancer Society survivorship recommendations [3].

The broader Lifestyle Technology in Cancer survey also included modules on mHealth app use, health and lifestyle behaviors, and social and structural determinants of health. These modules were collected to support a series of future analyses and manuscripts focused on different aspects of mHealth engagement; however, they fall outside the scope of the study. Here, the fitness-tracker module and its associated outcomes (ownership and use) are reported, consistent with the analytic aims of this study. All items were administered electronically in English via REDCap, with multiple-choice responses and optional free-text fields. The adapted survey was pilot-tested internally for clarity prior to study launch and can be found in Multimedia Appendix 1.

### Measures

The survey assessed multiple domains related to fitness tracker ownership, use, and perceptions. Participants were first asked whether they currently owned a fitness tracker and, if so, to indicate the device brand, duration of ownership, and frequency of wear. Follow-up questions explored reasons for using the tracker, including activity monitoring, sleep and heart rate tracking, goal setting, motivation, and accountability, as well as reasons for nonuse, such as discomfort, technical issues, or loss of interest. Non-owners were asked to identify reasons for not owning a tracker (eg, cost, lack of interest, privacy concerns, or feeling self-motivated without a device) and to indicate whether they were interested in obtaining one in the future. Barriers to and facilitators of use were assessed through multiple-choice items adapted from Holko et al [12], with optional free-text responses, allowing participants to elaborate on personal challenges and motivations related to wearable engagement.

Sociodemographic and social determinants of health variables were assessed using items adapted from the PRAPARE (Protocol for Responding to and Assessing Patient Assets, Risks, and Experiences) survey, a widely used, nationally standardized screening tool for social and structural determinants of health in clinical settings [13]. Psychosocial measures included perceived stress and confidence in one’s ability to take good care of one’s health. The confidence item was adapted from the Health Information National Trends Survey (HINTS-5), a nationally administered and validated population-based survey [2].

### Statistical Analyses

Descriptive statistics, including means (SEs) and percentages, were used to characterize participants and their reported reasons for owning and using a fitness tracker. To explore associations between various factors and fitness tracker ownership, univariate logistic regression was performed to estimate odds ratios (ORs) and 95% CIs for demographic, socioeconomic, psychosocial, and PA-related variables. Similarly, univariate logistic regression was used to assess associations between demographic characteristics and specific challenges that may hinder fitness tracker usage (eg, concerns about accuracy, discomfort, or lack of motivation). These associations were evaluated in relation to frequent use (ie, using a fitness tracker on most days or every day) versus less frequent use (including the remaining categories). This binary threshold was predetermined to differentiate habitual use from sporadic use, as both “most days” and “every day” signify a consistent integration of the device into daily life. Furthermore, this threshold was selected to align methodologically with the common operationalization of adherence in digital health and the wearable literature [14]. To identify independent predictors of fitness tracker ownership and frequent use, multivariable logistic regression models were constructed. Variables with at least marginal statistical significance (*P*<.15) in univariate analyses were initially included. A stepwise backward elimination approach was then applied: variables were removed sequentially, starting with the least significant, until all remaining variables were either (1) statistically significantly associated with the outcome or (2) demonstrated substantive confounding effects (defined as altering the association of another variable in the model with the outcome by ≥15%). The final multivariable models thus included only those factors that were independently associated with fitness tracker ownership or frequent use. Breast cancer was selected as the reference category for the cancer-site variable because it represented the largest single cancer site in the analytic sample (n=278, 31.1%) and provided the most stable and interpretable comparison group; this choice is descriptive only and does not imply a substantive hypothesis about breast cancer survivors as a normative referent. This variable selection strategy was chosen to accommodate the exploratory aims of the study, which sought to identify correlates of fitness tracker ownership and use across a broad set of candidate domains, for which no single established theoretical framework jointly specifies predictors in cancer survivor populations. A purely confirmatory, theory-driven specification was therefore not feasible, and a data-driven screening approach was selected to allow empirical identification of independently associated factors, while controlling for potential confounding. Multicollinearity among candidate predictors was evaluated using variance inflation factors (VIFs) prior to model fitting; all VIFs were below conventional thresholds (<5), indicating no problematic collinearity. To assess model stability, both multivariable models were refit with all candidate predictors entered simultaneously (Tables S1 and S2 in Multimedia Appendix 2) to confirm that the direction and magnitude of the primary associations were consistent across specifications. Given the cross-sectional design, all models estimated associations and could not establish temporal order or causality. Moreover, the two regression models were crafted with distinct analytical objectives. The ownership model explored a wide array of sociodemographic, clinical, and behavioral factors to pinpoint correlates linked to device ownership. In contrast, the frequent-use model was more narrowly focused, concentrating on modifiable usability and psychological barriers reported by device owners.

Notably, PA was excluded as a potential predictor in the frequent-use model due to this model’s emphasis on actionable usability and psychological barriers. Complete-case analysis was used for all regression models; participants with missing data on any variable included in a given model were excluded from that analysis. For household income, which had a substantial nonresponse rate (149/893, 16.7%), “no response” was retained as an explicit category in both univariable and multivariable ownership models rather than excluding these participants in order to preserve the sample size and avoid bias from listwise deletion of a nonrandomly missing variable. Missingness on other covariates was minimal and handled via complete-case exclusion. The multivariable model predicting fitness tracker ownership included 893 participants, and the multivariable model predicting frequent use among device owners included 504 participants.

## Results

### Participant Characteristics

**Figure 1:**
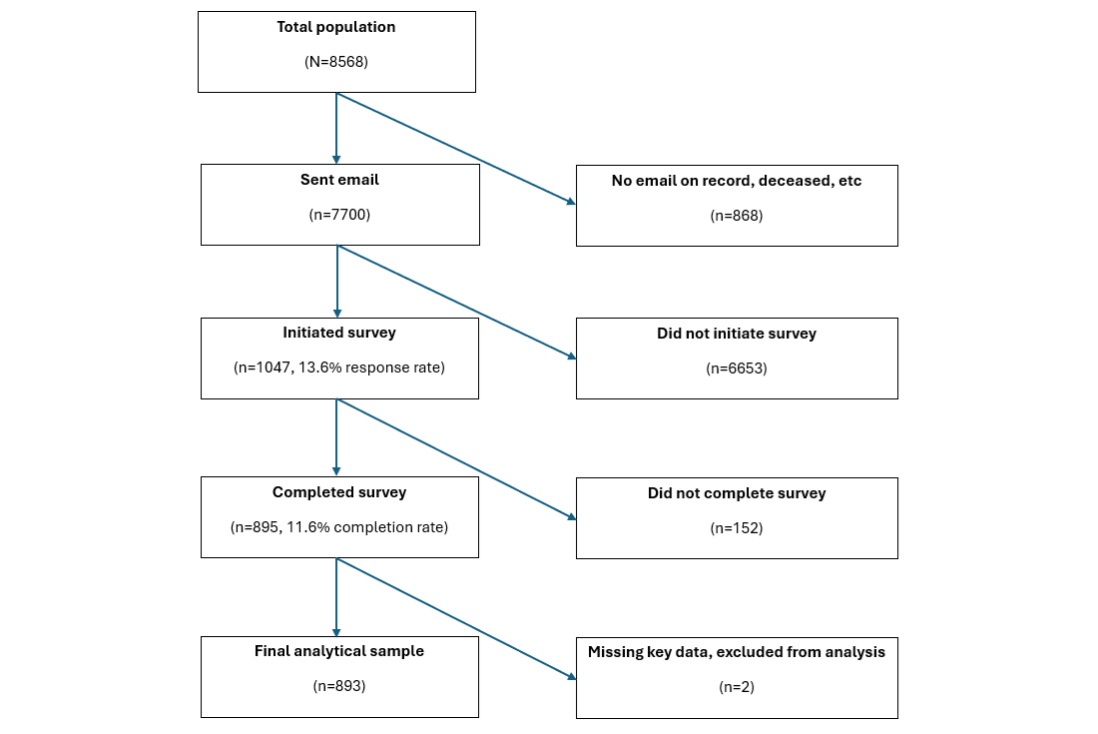
Participant selection flowchart based on CONSORT guidelines. CONSORT: Consolidated Standards of Reporting Trials.

Figure 1 shows the participant selection flowchart based on CONSORT guidelines. A total of 893 cancer survivors were included in the analytic sample (Table 1). The mean age was 61.8 (SE 0.38) years; 68.8% (614/893) identified as female and 93.5% (835/893) as non-Hispanic White. The most common cancer sites were breast (278/893, 31.1%), thyroid (166/893, 18.6%), and oral cavity/pharynx (149/893, 16.7%). The majority of survivors had the *International Classification of Diseases for Oncology* (ICD-O) cancer classification of “malignant” (742/893, 83.1%), and the mean time since diagnosis at the time of survey completion was 10.3 (SE 0.20) years. Approximately one-third of the participants reported annual household incomes exceeding US $100,000 (324/893, 36.3%), and 68.3% (610/893) earned at least a college degree. Regarding psychosocial factors, 20.8% (186/893) participants reported feeling “quite a bit” or “very much” stressed, and 52.3% (467/893) were “very” or “completely” confident in their ability to take good care of their health.

**Table 1:** Sample demographic and socioeconomic factors and responses to questions concerning stress and confidence in taking care of health for 893 mHealth^a^ participants.

Characteristics			Value
Age^b^ (years), mean (SE)			61.8 (0.38)
**Age group (years), n (%)**			
	<50	138 (15.5)	
	50-59	208 (23.3)	
	60-69	316 (35.4)	
	≥70	231 (25.9)	
**Sex, n (%)**			
	Female	614 (68.8)	
	Male	279 (31.2)	
**Race/ethnicity, n (%)**			
	White/non-Hispanic	835 (93.5)	
	Other	58 (6.5)	
**Cancer site, n (%)**			
	Breast	278 (31.1)	
	Thyroid	166 (18.6)	
	Oral cavity/pharynx	149 (16.7)	
	Bone marrow	61 (6.8)	
	All other sites	239 (26.8)	
**Malignancy level, n (%)**			
	Malignant	742 (83.1)	
	In situ	125 (14.0)	
	Benign/unknown	26 (2.9)	
Time since diagnosis (years), mean (SE)			10.3 (0.2)
**Time since diagnosis (years), n (%)**			
	<5	147 (16.5)	
	5-10	389 (43.6)	
	>10	357 (40.0)	
**Household income (US $), n (%)**			
	0-49,999	171 (19.1)	
	50,000-99,999	249 (27.9)	
	≥100,000	324 (36.3)	
	No response	149 (16.7)	
**Education, n (%)**			
	High school diploma/General Educational Development [GED] or less	109 (12.2)	
	Some college	169 (18.9)	
	College degree	359 (40.2)	
	Graduate/professional degree	251 (28.1)	
	No response	5 (0.6)	
**Stress level , n (%)**			
	Not at all	136 (15.2)	
	A little bit	313 (35.1)	
	Somewhat	254 (28.4)	
	Quite a bit/very much	186 (20.8)	
	No response	4 (0.4)	
**Confidence about ability to take good care of health, n (%)**			
	Completely confident	126 (14.1)	
	Very confident	341 (38.2)	
	Somewhat confident	316 (35.4)	
	A little confident/not confident at all	108 (12.1)	
	No response	2 (0.2)	

^a^mHealth: mobile health.

^b^Age is presented as a continuous variable with mean (SE). Percentages may not total 100% due to rounding. Small cell sizes (eg, <5) are presented because the data were fully deidentified and not linked to sensitive or identifiable subgroups. Categories with small response counts (eg, <5) should be interpreted with caution.

### Fitness Tracker Ownership

**Figure 2:**
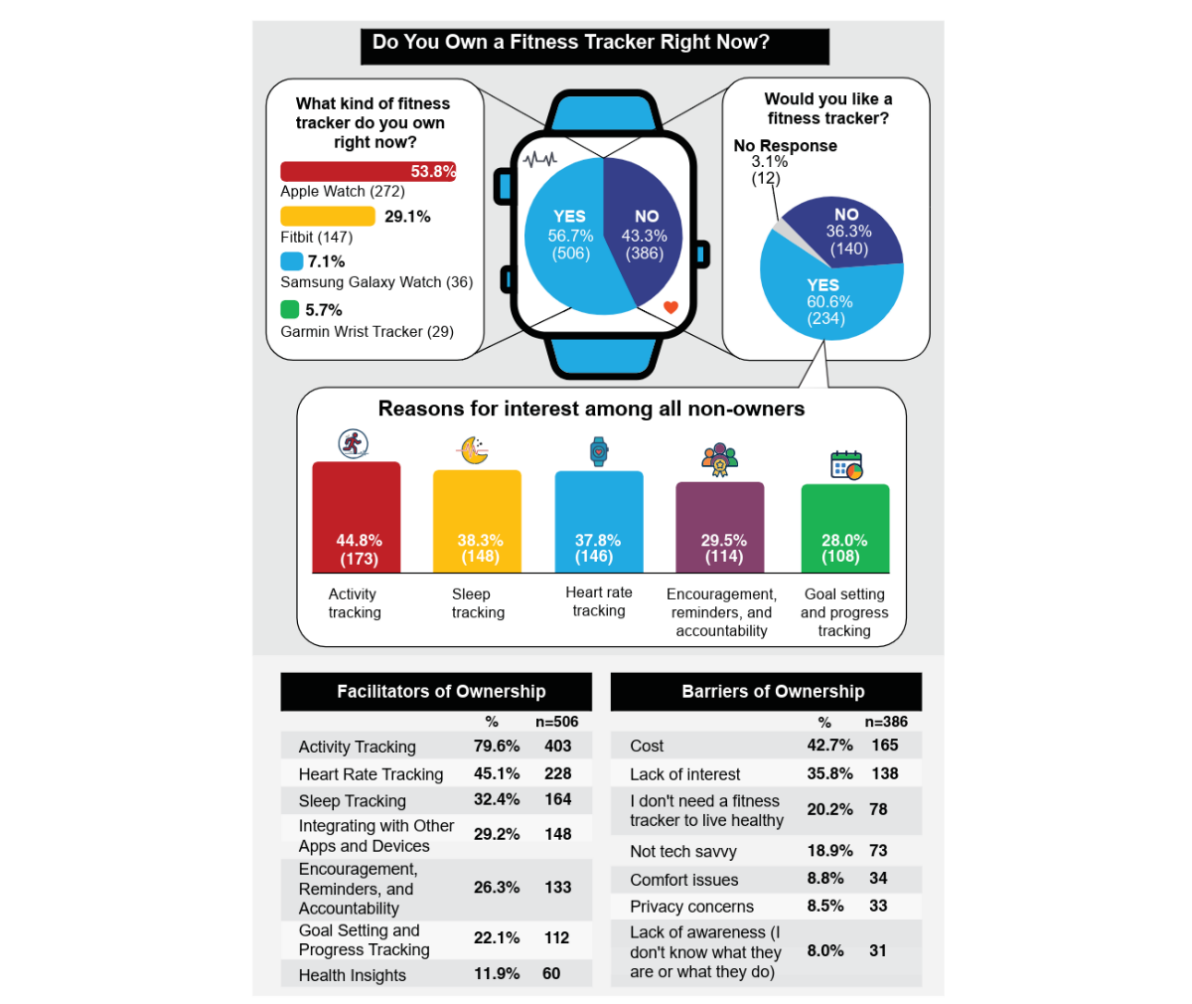
Fitness tracker ownership, interest, and associations among cancer survivors. Participants were asked about their current ownership of fitness trackers, their interest in using one, and specific features or functionalities they find helpful. Over half reported owning a tracker, with the Apple iWatch being most common. Among those interested in owning one, top motivations included activity tracking and health monitoring. Facilitators of ownership included usability features such as integration with other apps, while common barriers were cost and lack of interest. Participants could select multiple responses, but those who chose “no response” for a section could not select other options. The "%" column refers to the percentage of participants who selected each option. Participants could select multiple responses.

More than half of the respondents (506/893, 56.7%) reported currently owning a fitness tracker, while 43.3% (386/893) did not, and 1 (0.1%) individual declined to answer (Figure 2). The most common devices were the Apple iWatch (272/506, 53.8%), Fitbit (147/506, 29.1%), the Samsung Galaxy Watch (36/506, 7.1%), and the Garmin Wrist Tracker (29/506, 5.7%). Among non-owners, 60.6% (234/386) participants expressed interest in obtaining a tracker, with the most common reasons being to monitor activity (173/386, 44.8%), sleep (148/386, 38.3%), and heart rate (146/386, 37.8%), as well as for encouragement, reminders, and accountability (114/386, 29.5%).

The most frequently endorsed facilitators of ownership were activity tracking (403/506, 79.6%), heart rate tracking (228/506, 45.1%), sleep tracking (164/506, 32.4%), and integration with other apps and devices (148/506, 29.2%), followed by goal setting or progress tracking (112/506, 22.1%). Preventive health tracking (38/506, 7.5%) and food/calorie tracking (31/506, 6.1%) were less commonly reported. Barriers to ownership reported among non-owners included cost (165/386, 42.7%) and lack of interest (138/386, 35.8%), which were the most frequently cited reasons for not having a tracker. Other common barriers were believing they did not need a fitness tracker to stay healthy (78/386, 20.2%) and limited technology skills (73/386, 18.9%). Smaller proportions of participants reported comfort issues (34/386, 8.8%), privacy concerns (33/386, 8.5%), and lack of awareness about fitness trackers (31/386, 8.0%).

In univariate analyses (Table 2), several demographic and clinical factors were significantly associated with fitness tracker ownership. A younger age, female sex, a higher household income, higher educational attainment, and the cancer site were each associated with greater odds of ownership in unadjusted models. Specifically, male participants had lower odds of ownership compared to females (OR 0.42, 95% CI 0.31-0.56; *P*<.001), while higher household income was positively associated with ownership (US $50,000-$99,999: OR 1.61, 95% CI 1.08-2.38; *P*=.02; ≥US $100,000: OR 3.85, 95% CI 2.61-5.68; *P*<.001).

In the final adjusted multivariable model, fitness tracker ownership remained independently associated with sex, household income, and cancer site. Male participants had 39% lower odds of ownership compared to females (adjusted odds ratio [aOR] 0.61, 95% CI 0.42-0.87; *P*=.006). Higher household income was strongly associated with ownership, with participants reporting annual household incomes of US $50,000-$99,999 (aOR 1.70, 95% CI 1.13-2.57; *P*=.01) and ≥US $100,000 (aOR 3.64, 95% CI 2.41-5.50; *P*<.001) having greater odds of owning a tracker compared to those earning <US $50,000. Ownership also varied by cancer site, with lower odds observed among participants with thyroid cancer (aOR 0.64, 95% CI 0.41-1.00; *P*=.05), oral cavity/pharyngeal cancer (aOR 0.39, 95% CI 0.25-0.63; *P*<.001), bone marrow cancer (aOR 0.49, 95% CI 0.26-0.90; *P*=.02), and other cancer types (aOR 0.48, 95% CI 0.31-0.73; *P*<.001) relative to the reference group. Age was marginally associated with ownership in the adjusted model (aOR 0.99 per year, 95% CI 0.97-1.00; *P*=.05). Education, race/ethnicity, the BMI, perceived stress, and self-rated health confidence were not independently associated with ownership after adjustment.

The demographic homogeneity of the sample (primarily non-Hispanic White, college-educated individuals with higher household incomes, recruited via email) may limit statistical power to detect associations for certain characteristics, particularly race/ethnicity. Consequently, null findings for sociodemographic variables, such as race/ethnicity and education, should be interpreted in this context rather than as evidence that disparities do not exist. Finally, in a sensitivity analysis in which all candidate predictors were entered simultaneously, the direction, magnitude, and significance of associations were largely consistent with the reduced model (Table S1 in Multimedia Appendix 2).

**Table 2:** Logistic regression predicting fitness tracker ownership.

Variables		Univariable				Multivariable (n=893)^a^		
		OR^b^ (95% CI)	*P* value		aOR^c^ (95% CI)		*P* value	
Age (per year increase)		0.97 (0.96-0.98)		<.001		0.99 (0.97-1.00)		.052
**Sex**								
	Female	Reference	—^d^		Reference		—	
	Male	0.42 (0.31-0.56)^e^	<.001^e^		0.61 (0.42-0.87)^e^		.006	
**Race/ethnicity**								
	White, non-Hispanic	Reference	—		Reference		—	
	Other	0.77 (0.46-1.31)	.34		—		—	
**Annual household income (US $)**								
	0-49,999	Reference	—		—		—	
	50,000-99,999	1.61 (1.08-2.38)^e^	.02^e^		1.70 (1.13-2.57)^e^		.01	
	≥100,000	3.85 (2.61-5.68)^e^	<.001^e^		3.64 (2.41-5.50)^e^		<.001^e^	
	No response	1.36 (0.88-2.12)	.17		1.47 (0.93-2.33)		.10	
**Education**								
	High school diploma/General Educational Development (GED) or less	Reference	—		Reference		—	
	Some college	1.05 (0.65-1.70)	.85		—		—	
	College degree	2.13 (1.38-3.29)^e^	<.001^e^		—		—	
	Graduate/professional degree	1.85 (1.17-2.91)^e^	.008^e^		—		—	
**Cancer site**								
	Breast	Reference	—		—		—	
	Thyroid	0.63 (0.42-0.95)^e^	.03^e^		0.64 (0.41-1.00)^e^		.05^e^	
	Oral cavity/pharynx	0.27 (0.18-0.41)^e^	<.001^e^		0.39 (0.25-0.63)^e^		<.001^e^	
	Bone marrow	0.32 (0.18-0.56)^e^	<.001^e^		0.49 (0.26-0.90)^e^		.02^e^	
	All other sites	0.33 (0.23-0.47)^e^	<.001^e^		0.48 (0.31-0.73)^e^		<.001^e^	
**Perceived stress level**								
	Not at all	Reference	—		Reference		—	
	A little bit	1.23 (0.83-1.85)	.306		—		—	
	Somewhat	1.48 (0.98-2.26)	.07		—		—	
	Quite a bit/very much	1.43 (0.91-2.23)	.12		—		—	
**Confidence in self-care**								
	Completely confident	Reference	—		—		—	
	Very confident	0.84 (0.56-1.28)	.43		—		—	
	Somewhat confident	0.79 (0.52-1.20)	.26		—		—	
	A little/not at all confident	0.80 (0.47-1.34)	.39		—		—	
**PA^f^** **frequency**								
	Every day	Reference	—		—		—	
	4-5 days/week	0.93 (0.64-1.36)	.72		—		—	
	2-3 days/week	1.04 (0.70-1.54)	.85		—		—	
	<2 days/week	0.72 (0.49-1.05)	.09		—		—	
**BMI category (kg/m²)**								
	<25.0 (normal weight)	Reference	—		—		—	
	25.0-29.9 (overweight)	0.89 (0.63-1.26)	.51		—		—	
	≥30.0 (obese)	0.82 (0.59-1.14)	.24		—		—	

^a^The multivariable model included variables with *P*<.15 in univariable analyses; backward elimination retained variables that were statistically significant or demonstrated substantive confounding (≥15% change in another coefficient).

^b^OR: odds ratio.

^c^aOR: adjusted odds ratio.

^d^Variables not retained are indicated by an em dash (—). The univariable sample included all 893 participants; the multivariable model was fit on n=893. Variables included in the multivariable model were mutually adjusted.

^e^Statistical significance at *P*<.05 (two sided).

^f^PA: physical activity.

### Use Patterns and Perceived Barriers Among Fitness Tracker Owners

**Figure 3:**
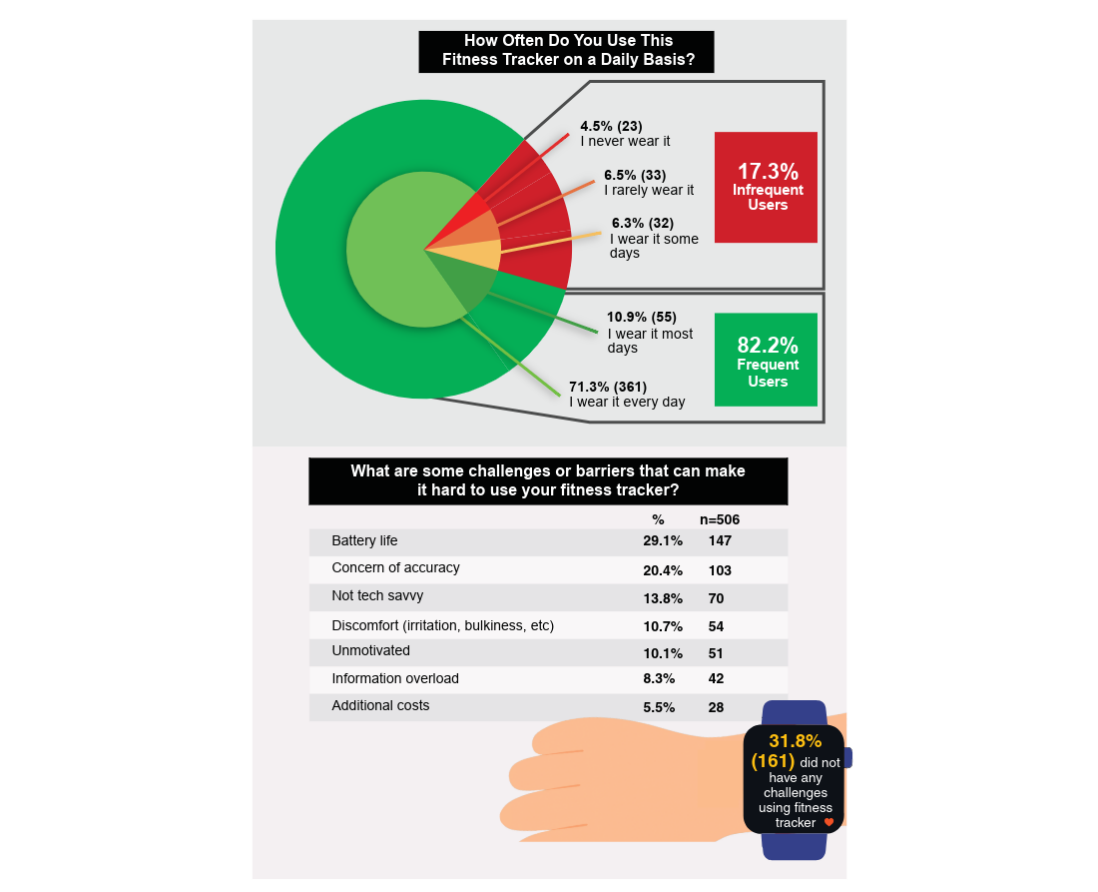
Frequency of fitness tracker use and barriers to use among cancer survivors. Participants were asked how often they used their fitness tracker and whether they experienced any challenges with use. Most participants reported wearing their tracker daily, while others indicated less frequent use. Of the five response categories, three reflected infrequent use, while two represented frequent use. Among the identified barriers, the most common were battery life, accuracy concerns, and discomfort. Participants could select more than one challenge. Barriers were only reported if participants either selected specific issues or indicated “I did not have any challenges.” Those who chose not to answer or skipped the question were excluded from the barrier summary. The "%" column refers to the percentage of participants who selected each option. Participants could select multiple responses. Those who chose “no response” for a section were not allowed to select additional options. Note: 0.5% of responses are missing due to nonresponse.

Among participants who owned a fitness tracker, most reported daily use: 71.3% (361/506) wore their device every day, and an additional 10.9% (55/506) wore it most days, while 6.3% (32/506) wore it some days, 6.5% (33/506) rarely used it, 4.5% (23/506) never used it, and 0.4% (2/506) declined to answer the question (Figure 3). For analytic purposes, “never,” “rarely,” and “some days” were grouped as infrequent use (88/506, 17.3%), and “most days” and “every day” were categorized as frequent use (416/506, 82.2%). This binary variable served as the outcome for the logistic regression analyses presented in Table 3.

Among current users, the most commonly reported challenges to regular use were battery life (147/506, 29.1%) and concerns about accuracy (103/506, 20.4%). Other barriers included not feeling tech savvy (70/506, 13.8%); discomfort, such as irritation or bulkiness (54/506, 10.7%); low motivation (51/506, 10.1%); information overload (42/506, 8.3%); and additional costs (28/506, 5.5%). Notably, nearly one-third of the owners (161/506, 31.8%) reported having no challenges using their device.

In multivariable models (Table 3), several barriers were significantly associated with lower odds of being a frequent tracker user. In the multivariable model, participants who reported device discomfort or irritation were 75% less likely to use their trackers frequently (aOR 0.25, 95% CI 0.13-0.48; *P*<.001). Those citing information overload were 55% less likely to use their trackers frequently (aOR 0.45, 95% CI 0.21-0.97; *P*=.04). Similarly, participants reporting low motivation were 76% less likely to use their trackers frequently (aOR 0.24, 95% CI 0.12-0.46; *P*<.001). Reports of device malfunction and lack of integration with other apps or devices were each associated with 75% lower odds of frequent use (aOR 0.25, 95% CI 0.10-0.62; *P*=.003; aOR 0.25, 95% CI 0.09-0.69; *P*=.007, respectively). Accuracy concerns, privacy issues, and battery life were not significantly associated with the frequency of use. It is important to note, however, that some barrier items (particularly low motivation and information overload) are conceptually proximate to the outcome of infrequent use and may partially reflect participants’ descriptions of their current behavior rather than independent antecedent factors. Device-level barriers, such as discomfort, malfunction, and limited app integration, are more clearly separable from use frequency, but the cross-sectional design precludes establishing temporal order for any of these associations. Finally, in a sensitivity analysis in which all candidate predictors were entered simultaneously, the direction and magnitude of the primary associations were consistent with the reduced model (Table S2 in Multimedia Appendix 2). Two variables near the conventional significance threshold exhibited minor shifts: concern about accuracy moved from nonsignificant to significant, and information overload moved from significant to nonsignificant. These fluctuations are consistent with the modest size of the infrequent-use group (n=88, 17.3%) that do not alter the substantive conclusions. A sensitivity analysis restricting the outcome to daily use produced largely consistent findings (Table S3 in Multimedia Appendix 2).

Overall, although most participants used their fitness trackers regularly, less frequent use was concurrently associated with self-reported device usability and motivational factors rather than demographic characteristics or privacy concerns. As these factors were measured at the same time as the outcome, they should be interpreted as correlates rather than established antecedent barriers.

**Table 3:** Logistic regression predicting frequent use^a^ of fitness trackers among current owners.

Variables		Univariable			Multivariable (n=504)^b^	
		OR^c^ (95% CI)	*P* value	aOR^d^ (95% CI)		*P* value
Age (per year increase)		1.00 (0.98-1.02)	.96	—^e^		—
**Sex**						
	Female	Reference	—	Reference		—
	Male	0.96 (0.59-1.58)	.88	—		—
**Reported barriers to use**						
	Accuracy concerns	1.77 (0.92-3.40)	.09	1.91 (0.96-3.78)		.07
	Discomfort (irritation, bulkiness)	0.30 (0.16-0.56)^f^	<.001^f^	0.25 (0.13-0.48)^f^		<.001^f^
	Not tech savvy	1.00 (0.53-2.00)	.94	—		—
	Battery life	0.65 (0.40-1.06)	.09	—		—
	Information overload	0.43 (0.22-0.87)^f^	.02^f^	0.45 (0.21-0.97)^f^		.04^f^
	Low motivation	0.27 (0.15-0.51)^f^	<.001^f^	0.24 (0.12-0.46)^f^		<.001^f^
	Privacy concerns	0.57 (0.18-1.84)	.35	—		—
	Additional costs	0.62 (0.25-1.50)	.28	—		—
	Limited app/device integration	0.24 (0.10-0.59)^f^	.002^f^	0.25 (0.09-0.69)^f^		.007^f^
	Device malfunction	0.33 (0.14-0.78)^f^	.01^f^	0.25 (0.10-0.62)^f^		.003^f^
	Limited internet access	0.56 (0.14-2.14)	.39	—		—
	No one to help me use it	0.91 (0.26-3.28)	.89	—		—
	Other	0.57 (0.18-1.84)	.35	—		—

^a^Frequent use was defined as wearing the device every day or most days (416/506, 82.2%); the reference category was infrequent use (rarely, some days, or never; 88/506, 17.3%).

^b^The multivariable model included variables with *P*<.15 in univariable analyses; backward elimination retained variables that were statistically significant or demonstrated substantive confounding (≥15% change in another coefficient).

^c^OR: odds ratio.

^d^aOR: adjusted odds ratio.

^e^Variables not retained are indicated by an em dash (—). All included variables were mutually adjusted.

^f^Statistical significance at *P*<.05 (two sided).

## Discussion

### Principal Findings

This study included one of the most comprehensive examinations to date of real-world fitness tracker ownership and engagement among cancer survivors. More than half of the respondents reported owning a tracker, yet frequent use (defined as wearing the device most days or every day) was less universal, highlighting important distinctions between device adoption and sustained engagement. Patterns of ownership were associated primarily with demographic and socioeconomic characteristics within this cohort, while continued use among device owners appeared more strongly related to psychosocial and usability-related factors. These prevalence estimates should be interpreted in light of the cohort’s characteristics: participants were recruited from a single institution, required an active email address to be contacted, and were predominantly non-Hispanic White, with a college education and a higher household income. Concurrent self-reports of device discomfort, information overload, and technical issues were associated with less consistent engagement in cross-sectional analysis.

Although the sample was relatively homogeneous in terms of race and ethnicity, which may limit the identification of certain access and equity-related barriers, the observed differences in ownership by sex, income, and cancer site highlight significant disparities in access, even within this group. Although this study was underpowered and not ideally suited to detect such patterns, they were still discernible within our limited sample. These patterns are becoming increasingly relevant as oncology moves toward digitally supported survivorship care, where fitness trackers offer a potential data source for monitoring PA, symptoms, and functional recovery.

### Comparison With Prior Research

In this study, approximately 57% of cancer survivors reported owning a fitness tracker, a prevalence higher than that reported in several prior population-based studies. This difference likely reflects, in part, the cohort’s recruitment approach and demographic composition rather than a uniform increase in adoption among cancer survivors generally. Despite this caveat, Zhou et al [15], using Health Information National Trends Survey – Surveillance, Epidemiology, and End Results (HINTS-SEER) data, documented increasing adoption of wearables among US cancer survivors over time, with ownership rising from approximately 21% in 2019 to 31% in 2022. Prior work has also documented lower wearable adoption among older cancer survivors. For example, Faro et al [16], in a study with a mean participant age of 74 years, found that only 31.8% of survivors owned a wearable device and that increasing age is inversely associated with ownership.

In addition to overall prevalence, we observed differences in fitness tracker ownership by sex, household income, and cancer site that are largely consistent with the established literature. Higher ownership among female participants corresponds with previous population-based research indicating a greater adoption of self-monitoring technologies by women. Similarly, higher ownership is also observed among individuals with higher incomes [17]. In this cohort, cancer-specific ownership patterns were also identified; however, such patterns have been less frequently explored in previous survivorship research, and the findings of this study should be viewed as preliminary observations to be tested in studies with detailed treatment and clinical data.

Population-based analyses of mHealth tracking have identified lower engagement among older cancer survivors. In a large HINTS-SEER analysis, Hong [18] reported that mHealth and behavior tracking is independently associated with younger age, although this outcome combines app- and wearable-based tracking rather than wearable ownership alone. In this study, age was significantly associated with fitness tracker ownership in unadjusted analyses and was marginally associated in adjusted models, suggesting that the age gradient in this cohort is partially but not fully explained by correlated sociodemographic factors. Differences in cohort composition, outcome definitions, and baseline levels of technology engagement may also contribute to variation across studies. Importantly, these findings should not be interpreted as evidence that age-related disparities have resolved, particularly in geriatric oncology populations, where continued evaluation remains warranted.

Prior oncology research has primarily evaluated the feasibility of fitness trackers under supervised conditions. In chemotherapy populations, Nilanon et al [19] demonstrated that continuous monitoring is feasible, with adherence above 68% and lower activity predicting unplanned clinical encounters. Ng et al [20] similarly reported very high adherence (94%-96%) and high perceived usefulness among inpatient and outpatient rehabilitation patients provided with trackers. These intervention-based findings confirm feasibility when devices are supported within structured settings, but reveal little about voluntary, sustained use. This study extended prior research by examining behavioral, motivational, and usability factors linked to real-world fitness tracker engagement.

### Interpretation of Key Findings

The observed associations between fitness tracker ownership and demographic and socioeconomic characteristics suggest that access to these technologies remains patterned by structural factors within cancer survivor populations. In this study, ownership was associated with household income, sex, and cancer site, indicating that socioeconomic resources and survivorship context may play a central role in shaping initial adoption. The income gradient is consistent with broader patterns of unequal access to consumer digital technologies documented in population-based studies [17]. Consumer-grade fitness trackers and smartwatches typically range from US $100 to US $400, a cost that may present a meaningful barrier for lower-income individuals, particularly given the financial burden associated with cancer treatment. Notably, cost was the most frequently endorsed barrier among non-owners in this study (42.7%), further supporting the interpretation that financial accessibility contributes to the observed income gradient.

Lower odds of fitness tracker ownership among male participants is consistent with sex-based differences in health self-monitoring behaviors observed in general population studies [17]. Prior research has documented that women are more likely than men to engage in preventive health behavior, patterns attributed, in part, to gender socialization around health vigilance and self-care [21].

Differences in fitness tracker ownership by cancer site have not been widely examined in prior survivorship research, and the cross-sectional design and absence of clinical covariates (treatment status, disease stage, recurrence, symptom burden, and functional limitations) preclude evaluation of the mechanisms underlying these patterns. The interpretations offered below are therefore hypothesis generating rather than explanatory. Breast cancer survivors, who served as the reference group, demonstrated the highest ownership prevalence, which may reflect both the demographic composition of this group (predominantly female, higher income) and the well-established culture of survivorship engagement, peer support, and wellness-oriented self-management in breast cancer communities. The lower ownership observed among survivors of oral cavity/pharynx cancers (aOR 0.39), bone marrow malignancies (aOR 0.49), and other sites may reflect differences in the treatment burden, physical sequelae that limit device wear (eg, peripheral neuropathy, dermatologic toxicity), or lower exposure to fitness-oriented survivorship messaging.

In contrast to ownership, frequent use among device owners was more closely associated with experiential and motivational factors within this cohort. Device discomfort, technical malfunctions, limited app integration, and information overload emerged as the most strongly associated barriers with lower frequent use. However, these associations warrant different levels of interpretive confidence. Device-level factors, such as discomfort, malfunction, and limited integration, are conceptually distinct from use frequency and are therefore more defensible as independent correlates. In contrast, self-reported motivational and cognitive burden items (low motivation, information overload) overlap conceptually with the outcome itself: reporting that one is “unmotivated” to use a tracker is difficult to disentangle from the behavior of not using it. These items may therefore function, in part, as retrospective explanations for disengagement. This distinction does not invalidate the observed associations but does constrain their interpretation; none should be treated as established causal barriers, given the cross-sectional design. With this caveat, our findings align with prior digital oncology research emphasizing simplicity, interoperability, and actionable feedback as key correlates of sustained engagement [10,11].

Fitness trackers play a key role in PA monitoring, a central behavioral target in survivorship. PA is safe and beneficial before, during, and after treatment, improving fatigue, functional capacity, and quality of life and reducing mortality risk [6,7,22]. In a systematic review, Singh et al [9] found moderate-to-large improvements in PA and related outcomes from wearable-supported interventions. Similarly, oncology rehabilitation frameworks emphasize early identification of functional decline and referral to exercise oncology services [7]. Together, these bodies of work highlight why understanding real-world fitness tracker engagement is clinically significant.

### Implications for Digital Health and Survivorship Care

Although this study did not evaluate implementation strategies, clinical integration models, or interventions targeting usability, the observed patterns of fitness tracker ownership and use may inform future research and program development. As survivorship care increasingly incorporates remote monitoring, the relatively high prevalence of fitness tracker ownership in this cohort (506/893, 56.7%) and frequent use among owners (416/506, 82.2%) suggest that for digitally engaged subgroups, wearables could meaningfully extend assessment beyond the clinic. These rates should not, however, be taken as population-level estimates of what scalable tracker-enabled care can rely on. Wearables may support the identification of emerging functional vulnerability by enabling longitudinal monitoring of mobility-related activity patterns, including sustained reductions in daily step counts and overall activity levels, which have been associated with postoperative recovery trajectories, readmissions, and frailty-related outcomes in cancer and older adult populations [23-25]. In parallel, wearable-derived measures of PA, sedentary behavior, heart rate, and sleep have been proposed as scalable digital indicators of cardiovascular and cardiometabolic risk in cancer survivorship, complementing clinic-based evaluations rather than replacing them [26].

The significant associations between sociodemographic factors and device ownership indicate that equitable access may be the first and most critical step in facilitating fitness tracker adoption. The strong income gradient observed in this study, combined with cost being the most frequently endorsed barrier among non-owners, suggests that financial accessibility remains a meaningful obstacle for lower-income participants. Strategies to address this barrier may include device subsidy or loaner programs, integration of fitness tracker provision into survivorship care plans, or partnerships with device manufacturers to reduce cost. Similarly, the lower ownership observed among male participants and among survivors of certain cancer types suggests that outreach and engagement strategies may need to be tailored to reach populations that are less likely to adopt these technologies independently.

However, inconsistent engagement among device owners highlights that access alone may not be sufficient to support sustained use. Future studies should evaluate implementation approaches that directly address behavioral and usability barriers, including device comfort, cognitive burden, and platform interoperability. Evidence from patients receiving care at federally qualified health centers indicates that baseline wearable ownership is low (21%), despite substantial interest among those without a device (58%), highlighting that effective implementation may require education, onboarding, and user support to address commonly reported usability and knowledge barriers beyond device provision alone, even when economic barriers are addressed [12]. It is important to note, however, that the ownership prevalence in this study cohort was significantly higher, at 56.7%, and the cohort itself showed limited sociodemographic diversity. This suggests that further research involving more diverse study populations is necessary to determine if the sociodemographic patterns observed within this study generalize to populations with greater racial, ethnic, and socioeconomic diversity.

Finally, because most owners in this cohort were already using consumer devices, such as the Apple iWatch and Fitbit, future digital lifestyle interventions may benefit from supporting multidevice integration and bring-your-own-device approaches, allowing cancer survivors to continue using familiar technologies and reducing friction to participation. Evaluating these strategies in more diverse populations and health care settings will be essential to determine how tracker-enabled survivorship approaches can be implemented effectively and equitably.

### Strengths and Limitations

This study has several limitations. The cross-sectional design precludes causal inference, and fitness tracker ownership and use were self-reported rather than objectively verified, which may introduce recall or social desirability bias. Second, the dataset lacked clinical characteristics, such as treatment status and recurrence history, which may limit interpretation. In addition, in the use-frequency analysis (Table 3), both the barrier items and the outcome were ascertained simultaneously through the same self-report instrument. Although device-level barriers (eg, discomfort, malfunction) are conceptually separable from use frequency, motivational and cognitive burden items (eg, low motivation, information overload) are difficult to distinguish from the outcome they are intended to predict, raising the possibility of criterion contamination. Future longitudinal designs that assess barriers at baseline and track use patterns prospectively would help clarify whether these factors precede, accompany, or result from changes in engagement.

The sample primarily consisted of non-Hispanic White individuals with a college educated and higher household incomes, which limits the generalizability to more diverse survivor populations. Although several sociodemographic correlates of ownership were detected, including sex, income, and cancer site, the limited racial and ethnic diversity of the sample constrains the ability to identify disparities along these dimensions, and the observed associations may not generalize to populations with greater sociodemographic variability. We were also unable to evaluate device-level characteristics, such as accuracy, brand-specific differences, or wear time verification, which constrain the interpretation of how technical performance may influence engagement. In particular, several of the barriers identified in the frequent-use model, such as battery life, device malfunction, and limited app or device integration, are plausibly device or brand specific and may vary by manufacturer, device generation, or operating-system ecosystem; because brand-level subgroup analyses were not powered in the sample, these barriers should be interpreted as aggregate user experiences rather than properties of fitness trackers as a class. Finally, although the survey captured additional digital health constructs, the analysis focuses specifically on fitness tracker behaviors to maintain conceptual and analytic clarity.

Despite these limitations, this study has important strengths. The sample included survivors across a broad range of cancer types, and the survey simultaneously assessed demographic, psychosocial, and experiential correlates of fitness tracker use, domains that are not often examined together. By characterizing real-world engagement with consumer-owned devices rather than study-provided fitness trackers, this work provides insight into naturalistic patterns of adoption and sustained use. These findings help fill a critical gap in the literature, which has largely been divided between population-level estimates of device ownership and feasibility studies conducted under structured clinical or research conditions. By integrating both perspectives, this study advances understanding of how survivors adopt and maintain fitness tracker use in everyday contexts, providing a behavioral foundation for developing scalable, equitable digital health interventions to support long-term self-management.

### Conclusion

In summary, we conducted one of the largest studies of real-world fitness tracker engagement among cancer survivors and found that within this single-center, demographically homogeneous cohort, although ownership was common, initial ownership was primarily correlated with sociodemographic factors, while sustained use within this sample varied widely and was concurrently associated with self-reported psychosocial and usability factors, which should be interpreted as correlates rather than established antecedent barriers. Because the sample likely reflects a digitally engaged subgroup, these patterns should be interpreted as upper-bound estimates rather than broadly representative figures. These findings highlight the importance of integrating behavioral support, human-centered design, and seamless system-level interoperability into digital survivorship programs. Future research should examine longitudinal associations between fitness tracker–derived activity and clinical outcomes, test interventions targeting motivational and emotional barriers, and develop implementation models that facilitate integration of fitness tracker data into oncology workflows. By strengthening both access and sustained engagement, fitness trackers may help support scalable, personalized approaches to survivorship care.

## Appendix

Multimedia Appendix 1 Survey instruments.

Multimedia Appendix 2 Regression sensitivity analysis.

## Abbreviations

aOR: adjusted odds ratio
CONSORT: Consolidated Standards of Reporting Trials
HINTS-SEER: Health Information National Trends Survey – Surveillance, Epidemiology, and End Results
mHealth: mobile health
OR: odds ratio
PA: physical activity
REDCap: Research Electronic Data Capture
TCC: Total Cancer Care
VIF: variance inflation factor

## Data Availability

The statistical code used to analyze the data in this study is available from the corresponding author upon reasonable request. The datasets generated or analyzed during this study are not publicly available as they contain confidential patient information but can be made available from the corresponding author upon reasonable request.

## References

[1] Gresham G, Schrack J, Gresham LM, Shinde AM, Hendifar AE, Tuli R, Rimel BJ, Figlin R, Meinert CL, Piantadosi S (2018). Wearable activity monitors in oncology trials: current use of an emerging technology. Contemp Clin Trials.

[2] Benzo R, Tetrick M, Krok-Schoen J, Brasky T, Washington P, Paskett E, Penedo F, Singh R, Shechtman M, Fernandez S, Kumar S, Mallahzadeh M, Fisher JL (2025). Digital information-seeking behaviors among cancer survivors: associations with sociodemographic determinants, cancer history, and perceived health. J Cancer Surviv.

[3] Rock CL, Thomson CA, Sullivan KR, Howe CL, Kushi LH, Caan BJ, Neuhouser ML, Bandera EV, Wang Y, Robien K, Basen-Engquist KM, Brown JC, Courneya KS, Crane TE, Garcia DO, Grant BL, Hamilton KK, Hartman SJ, Kenfield SA, Martinez ME, Meyerhardt JA, Nekhlyudov L, Overholser L, Patel AV, Pinto BM, Platek ME, Rees-Punia E, Spees CK, Gapstur SM, McCullough ML (2022). American Cancer Society nutrition and physical activity guideline for cancer survivors. CA Cancer J Clin.

[4] Campbell KL, Winters-Stone KM, Wiskemann J, May AM, Schwartz AL, Courneya KS, Zucker DS, Matthews CE, Ligibel JA, Gerber LH, Morris GS, Patel AV, Hue TF, Perna FM, Schmitz KH (2019). Exercise guidelines for cancer survivors: consensus statement from International Multidisciplinary Roundtable. Med Sci Sports Exerc.

[5] McTiernan A, Friedenreich CM, Katzmarzyk PT, Powell KE, Macko R, Buchner D, Pescatello LS, Bloodgood B, Tennant B, Vaux-Bjerke A, George SM, Troiano RP, Piercy KL (2019). Physical activity in cancer prevention and survival: a systematic review. Med Sci Sports Exerc.

[6] Schmitz KH, Campbell AM, Stuiver MM, Pinto BM, Schwartz AL, Morris GS, Ligibel JA, Cheville A, Galvão DA, Alfano CM, Patel AV, Hue T, Gerber LH, Sallis R, Gusani NJ, Stout NL, Chan L, Flowers F, Doyle C, Helmrich S, Bain W, Sokolof J, Winters-Stone KM, Campbell KL, Matthews CE (2019). Exercise is medicine in oncology: engaging clinicians to help patients move through cancer. CA Cancer J Clin.

[7] Stout NL, Brown JC, Schwartz AL, Marshall TF, Campbell AM, Nekhlyudov L, Zucker DS, Basen-Engquist KM, Campbell G, Meyerhardt J, Cheville AL, Covington KR, Ligibel JA, Sokolof JM, Schmitz KH, Alfano CM (2020). An exercise oncology clinical pathway: screening and referral for personalized interventions. Cancer.

[8] Chow R, Drkulec H, Im JHB, Tsai J, Nafees A, Kumar S, Hou T, Fazelzad R, Leighl NB, Krzyzanowska M, Wong P, Raman S (2024). The use of wearable devices in oncology patients: a systematic review. Oncologist.

[9] Singh B, Zopf EM, Howden EJ (2022). Effect and feasibility of wearable physical activity trackers and pedometers for increasing physical activity and improving health outcomes in cancer survivors: a systematic review and meta-analysis. J Sport Health Sci.

[10] Keats M, Yu X, Sweeney Magee M, Forbes C, Grandy S, Sweeney E, Dummer T (2023). Use of wearable activity-monitoring technologies to promote physical activity in cancer survivors: challenges and opportunities for improved cancer care. Int J Environ Res Public Health.

[11] Kos M, Brouwer CG, van Laarhoven HW, Hopman MT, van Oijen MG, Buffart LM (2023). The association between wearable device metrics and clinical outcomes in oncology: a systematic review with evidence synthesis and meta-analysis. Crit Rev Oncol Hematol.

[12] Holko M, Litwin TR, Munoz F, Theisz KI, Salgin L, Jenks NP, Holmes BW, Watson-McGee P, Winford E, Sharma Y (2022). Wearable fitness tracker use in federally qualified health center patients: strategies to improve the health of all of us using digital health devices. NPJ Digit Med.

[13] Wan W, Li V, Chin MH, Faldmo DN, Hoefling E, Proser M, Weir RC (2022). Development of PRAPARE social determinants of health clusters and correlation with diabetes and hypertension outcomes. J Am Board Fam Med.

[14] Miyaji T, Kawaguchi T, Azuma K, Suzuki S, Sano Y, Akatsu M, Torii A, Kamimura T, Ozawa Y, Tsuchida A, Eriguchi D, Hashiguchi M, Nishino M, Nishi M, Inadome Y, Yamazaki T, Kiuchi T, Yamaguchi T (2020). Patient-generated health data collection using a wearable activity tracker in cancer patients-a feasibility study. Support Care Cancer.

[15] Zhou W, Cho Y, Pu J, Shang S (2024). Trends in wearable device use among cancer survivors in the United States from 2019 to 2022. J Geriatr Oncol.

[16] Faro JM, Yue K, Singh A, Soni A, Ding EY, Shi Q, McManus DD (2022). Wearable device use and technology preferences in cancer survivors with or at risk for atrial fibrillation. Cardiovasc Digit Health J.

[17] Nagappan A, Krasniansky A, Knowles M (2024). Patterns of ownership and usage of wearable devices in the United States, 2020-2022: survey study. J Med Internet Res.

[18] Hong YA (2024). Mobile health and behavior tracking (mHBT) among cancer survivors: results from a large and diverse sample. Mhealth.

[19] Nilanon T, Nocera LP, Martin AS, Kolatkar A, May M, Hasnain Z, Ueno NT, Yennu S, Alexander A, Mejia AE, Boles RW, Li M, Lee JSH, Hanlon SE, Cozzens Philips FA, Quinn DI, Newton PK, Broderick J, Shahabi C, Kuhn P, Nieva JJ (2020). Use of wearable activity tracker in patients with cancer undergoing chemotherapy: toward evaluating risk of unplanned health care encounters. JCO Clin Cancer Inform.

[20] Ng A, Gupta E, Bansal S, Fontillas RC, Amos CE, Williams JL, Dibaj S, Bruera E (2021). Cancer patients' perception of usefulness of wearable exercise trackers. PM R.

[21] Deeks A, Lombard C, Michelmore J, Teede H (2009). The effects of gender and age on health related behaviors. BMC Public Health.

[22] Courneya KS, Booth CM (2022). Exercise as cancer treatment: a clinical oncology framework for exercise oncology research. Front Oncol.

[23] Jonker LT, Lahr MMH, Oonk MHM, de Bock GH, van Leeuwen BL (2021). Post-discharge telemonitoring of physical activity, vital signs, and patient-reported symptoms in older patients undergoing cancer surgery. Ann Surg Oncol.

[24] de Leeuwerk ME, Botjes M, van Vliet V, Geleijn E, de Groot V, van Wegen E, van der Schaaf M, Tuynman J, Dickhoff C, van der Leeden M (2022). Self-monitoring of physical activity after hospital discharge in patients who have undergone gastrointestinal or lung cancer surgery: mixed methods feasibility study. JMIR Cancer.

[25] Friedrich B, Elgert L, Eckhoff D, Bauer JM, Hein A (2023). A system for monitoring the functional status of older adults in daily life. Sci Rep.

[26] Benzo RM, Gogineni A, Tetrick MK, Singh R, Washington P, Fernandez S, Paskett ED, Penedo FJ, Ghazi S, Osei A, Clinton SK, Krok-Schoen J, Weyrauch S, Addison D (2025). mHealth technologies in research studying cardiovascular health in cancer: a systematic review. PLOS Digit Health.

