# Supplementary material for: Correlates of Fitness Tracker Ownership and Use in Cancer Survivors: Cross-Sectional Survey
Source: JMIR Cancer. 2026 May 26;12:e92876. doi: 10.2196/92876 (PMC13250496; doi:10.2196/92876)
Supplement: Multimedia Appendix 1 [file cancer_v12i1e92876_app1.pdf]

# Fitness Trackers

The following questions will ask about your experience, usage, and interests in fitness trackers.

Fitness trackers are electronic devices that can be worn as accessories, and most commonly in the form of smart watches like a Fitbit or Apple watch. They can be used to track or improve health behaviors like sleep, activity, and sedentary behavior.

Feel free to select "I choose not to answer this question" for any questions that you do not wish to answer.

Progress: 0%

| | |
|---|---|
| Did you know what a fitness tracker was before this survey? | ◯ No<br>◯ Yes<br>◯ I choose not to answer this question |
| Do you own a fitness tracker right now? | ◯ No<br>◯ Yes<br>◯ I choose not to answer this question |
| What kind of fitness tracker do you own right now? (Select all that apply) | ☐ Fitbit<br>☐ Apple watch<br>☐ Garmin wrist tracker<br>☐ Whoop wrist tracker<br>☐ Oura ring<br>☐ Amazfit wrist tracker<br>☐ Samsung Galaxy watch<br>☐ Google Pixel watch<br>☐ Other (please specify): ______<br>☐ I choose not to answer this question |
| How often do you use this fitness tracker on a daily basis? | ◯ I wear it every day<br>◯ I wear it most days<br>◯ I wear it some days<br>◯ I rarely wear it<br>◯ I never wear it<br>◯ I choose not to answer this question |
| Why did you buy this fitness tracker? (Select all that apply) | ☐ Activity tracking<br>☐ Heart rate tracking<br>☐ Preventative health tracking<br>☐ Sleep tracking<br>☐ Food/calorie tracking<br>☐ Goal setting and progress tracking<br>☐ Encouragement, reminders, and accountability<br>☐ Workout guidance<br>☐ Health insights<br>☐ Integrating with other apps and devices<br>☐ Social support and community<br>☐ Other: ______<br>☐ I choose not to answer this question |

What are some challenges or barriers that can make it hard to use your fitness tracker? (Select all that apply)

- ☐ Concern of accuracy
- ☐ Discomfort (irritation, bulkiness, etc.)
- ☐ Not tech savvy
- ☐ Battery life
- ☐ Information overload
- ☐ Unmotivated
- ☐ Privacy concerns
- ☐ Additional costs
- ☐ Does not integrate with other apps and devices
- ☐ Malfunction issues
- ☐ Language barrier
- ☐ Limited internet access
- ☐ Limited data plan on cell phone
- ☐ I have no one to help me use it
- ☐ Other (please specify): ______
- ☐ I do not have any challenges using my fitness tracker
- ☐ I choose not to answer this question

---

Why don't you currently have a fitness tracker? (Select all that apply)

- ☐ Cost
- ☐ Lack of interest
- ☐ Privacy concerns
- ☐ Comfort issues
- ☐ Concern of accuracy
- ☐ Lack of awareness (I don't know what they are or what they do)
- ☐ Not tech savvy
- ☐ Unmotivated to improve health behaviors
- ☐ I don't need a fitness tracker to live healthy (self-motivated)
- ☐ Limited internet access
- ☐ I don't have a smartphone
- ☐ Limited data plan on cell phone
- ☐ I have no one to help me use it
- ☐ Language barrier
- ☐ Other (please specify): ______
- ☐ I choose not to answer this question

---

Would you like a fitness tracker?

- ◯ No
- ◯ Yes
- ◯ I choose not to answer this question

---

If yes, why would you like a fitness tracker? (Select all that apply)

- ☐ Activity tracking
- ☐ Heart rate tracking
- ☐ Sleep tracking
- ☐ Food tracking
- ☐ Preventative health tracking
- ☐ Goal setting and progress tracking
- ☐ Encouragement, reminders, and accountability
- ☐ Workout guidance
- ☐ Health insights
- ☐ Integrating with other apps and devices
- ☐ Social support and community
- ☐ Other: ______
- ☐ I choose not to answer this question

| | |
|---|---|
| Have you ever owned a fitness tracker before? (Select all that apply) | ☐ No<br>☐ Yes (a Fitbit)<br>☐ Yes (an Apple watch)<br>☐ Yes (a Whoop)<br>☐ Yes (an Oura ring)<br>☐ Yes (an Amazfit wrist tracker)<br>☐ Yes (a Samsung Galaxy watch)<br>☐ Yes (a Google Pixel watch)<br>☐ Yes (Other: ______<br>☐ I choose not to answer this question |
| Would you recommend a fitness tracker to others? | ◯ No<br>◯ Yes<br>◯ I choose not to answer this question |
| Do you think fitness trackers can give you helpful information about your health? | ◯ No<br>◯ Yes<br>◯ I choose not to answer this question |
| Could you tell us why you chose this answer? | ______________________________ |
| If you have a fitness tracker (or if we gave you one), would you share the data with researchers?<br><br>(Data could include health measures like heart rate, step count, standing hours, etc.) | ◯ No<br>◯ Yes, and I have one<br>◯ Yes, if you give me one<br>◯ I choose not to answer this question |
| Would you like to learn how fitness trackers can help with your health? | ◯ No<br>◯ Yes<br>◯ I choose not to answer this question |
| Knowing that we are looking for ways to use fitness trackers to improve your health, is there anything else you want to tell us about fitness tracker use? | ______________________________ |

# Health and Lifestyle Assessments

The following questions will ask about the services you've received related to nutrition and physical activity since your cancer diagnosis, your health, and your thoughts on a digital lifestyle program.

Feel free to select "I choose not to answer this question" for any questions that you do not wish to answer.

Progress: 60%

| | |
|---|---|
| Since your diagnosis, have you had any physical activity assessment and counseling? | ◯ No<br>◯ Yes<br>◯ I choose not to answer this question |
| [Physical Activity]<br><br>Who did you see? (Select all that apply) | ☐ Exercise Physiologist<br>☐ Occupational Therapist<br>☐ Physical Therapist<br>☐ Personal Trainer<br>☐ Other (please specify): ______<br>☐ I don't know<br>☐ I choose not to answer this question |
| Since your diagnosis, have you had any nutritional assessment and counseling? | ◯ No<br>◯ Yes<br>◯ I choose not to answer this question |
| [Nutritional]<br><br>Who did you see? (Select all that apply) | ☐ Clinical Nutritionist<br>☐ Registered Dietetic Technician (DTR or NDTR)<br>☐ Registered Dietitian<br>☐ Public Health Nutritionist<br>☐ Nutrition Consultant<br>☐ Other (please specify): ______<br>☐ I don't know<br>☐ I choose not to answer this question |
| How often do you get 30 minutes or more of physical activity (such as walking, running, riding a bike)? | ◯ Every day<br>◯ 4-5 days per week<br>◯ 2-3 days per week<br>◯ Less than 2 days per week<br>◯ Never<br>◯ I choose not to answer this question |
| Overall, how confident are you about your ability to take good care of your health? | ◯ Completely confident<br>◯ Very confident<br>◯ Somewhat confident<br>◯ A little confident<br>◯ Not confident at all<br>◯ I choose not to answer this question |
| From the set of values below, which ONE is most important to you in your day-to-day life? | ◯ Making my own decisions<br>◯ Being happy<br>◯ Helping people<br>◯ Being loyal to family and friends<br>◯ Having a deep connection to my religion<br>◯ Keeping myself in good health<br>◯ Assuring my family is safe and secure<br>◯ Other (please specify): ______<br>◯ I choose not to answer this question |

**At the Lifestyle Lab, we are committed to enhancing lives through positive lifestyle behaviors. We recognize that each person has unique needs and preferences. So, the following questions are to better understand how we can tailor our work to support you.**

What is your preferred format for a lifestyle program? (Select all that apply)

- ☐ In-person
- ☐ Hybrid (in-person and virtual)
- ☐ Digital: Website (computer or tablet accessible)
- ☐ Digital: App-Based
- ☐ I choose not to answer this question

We are creating digital resources for cancer survivors and families.

What topics would you find most useful? (Select all that apply)

- ☐ Lifestyle recommendations for cancer prevention and survivors
- ☐ Workout videos: Fun ways to stay active and fit
- ☐ Building healthy habits
- ☐ Goal Setting
- ☐ Lifestyle strategies for managing common symptoms
- ☐ Tips for better sleep
- ☐ Coping with stress and staying positive
- ☐ Building helpful social connections
- ☐ Boosting energy levels
- ☐ Healthy eating tips for everyday life
- ☐ Other: ______
- ☐ I choose not to answer this question

If we made a digital lifestyle program (like an app or website) to improve your health behaviors... What features would you find most useful? (Select all that apply)

- ☐ Personalized Goal Setting
- ☐ Activity Tracking
- ☐ Online workouts
- ☐ Health coaching
- ☐ Sleep Tracking
- ☐ Sleep Environment Optimization
- ☐ Reminders and Notifications
- ☐ Progress Reports and Insights
- ☐ Integration with Wearable Devices
- ☐ Social Integration and Community Support
- ☐ Educational Resources
- ☐ Challenges and Rewards
- ☐ Virtual (AI) Coaching and Support
- ☐ Sharing data with Health Professionals
- ☐ Symptom and Mood Tracking
- ☐ Mindfulness and Meditation Exercises
- ☐ Stress Management Tools
- ☐ Food Tracking
- ☐ Healthy Recipes and Meal Plans
- ☐ Other: ______
- ☐ I choose not to answer this question

# Demographics

The following questions will allow us to learn more about you and your background.

Feel free to select "I choose not to answer this question" for any questions you do not wish to answer.

Progress: 70%

Age: ______________________

What is your sex assigned at birth?
- ◯ Male
- ◯ Female
- ◯ Intersex
- ◯ I choose not to answer this question

What gender do you identify with?
- ◯ Man
- ◯ Woman
- ◯ Genderqueer
- ◯ Transman
- ◯ Transwoman
- ◯ Non-binary
- ◯ Two-spirit
- ◯ Other: ______
- ◯ I choose not to answer this question

Height Feet: Inches:
______ ______

______________________

What is your weight (in pounds)? ______________________

BMI: ______________________

# Social Determinants of Health

The following questions will ask about your social determinants of health.

Social determinants of health are the conditions in which people are born, live, learn, work, play, worship, and age. Understanding your social determinants of health are important because these conditions affect a wide range of health, functioning, and quality-of-life outcomes and risks.

Feel free to select "I choose not to answer this question" for any questions that you do not wish to answer.

Progress: 80%

| | |
|---|---|
| Are you Hispanic or Latino? | ◯ No<br>◯ Yes<br>◯ I choose not to answer this question |
| Which race(s) do you identify as?  (Select all that apply) | ☐ Asian<br>☐ Native Hawaiian<br>☐ Pacific Islander<br>☐ Black/African American<br>☐ White<br>☐ American Indian/ Alaskan Native<br>☐ Other (please write): ______<br>☐ I choose not to answer this question |
| What language are you most comfortable speaking? | ◯ English<br>◯ Spanish<br>◯ Mandarin<br>◯ Other<br>◯ I choose not to answer this question |
| What language are you most comfortable speaking? Please write. | __________________________________ |
| How many family members, including yourself, do you currently live with? | ◯ 1 (just yourself)<br>◯ 2<br>◯ 3<br>◯ 4<br>◯ 5<br>◯ 6<br>◯ 7<br>◯ 8<br>◯ 9<br>◯ 10 or more<br>◯ I choose not to answer this question |
| What is your housing situation today? | ◯ I do not have housing (staying with others, in a hotel, in a shelter, living outside on the street, etc.)<br>◯ I have housing, I rent<br>◯ I have housing, I own<br>◯ I have housing, other<br>◯ I choose not to answer this question |
| Are you worried about losing your housing? | ◯ No<br>◯ Yes<br>◯ I choose not to answer this question |

| | |
|---|---|
| Address: (If you would, please provide your address number and street here) | __________________________ |
| Address: (Zip code here) | __________________________ |
| What is the highest level of school that you have finished? | ◯ Less than high school degree<br>◯ High school diploma or GED<br>◯ More than high school, some college<br>◯ College degree or more<br>◯ Graduate or professional degree<br>◯ I choose not to answer this question |
| What is your current work situation? | ◯ Unemployed<br>◯ Part-time or temporary work<br>◯ Full-time work<br>◯ Otherwise unemployed but not seeking work (ex: student, retired, disabled, unpaid primary caregiver) Please write: ______<br>◯ I choose not to answer this question |
| What is your main insurance? | ◯ None/uninsured<br>◯ Medicaid<br>◯ Medicare<br>◯ Other public insurance<br>◯ Private insurance<br>◯ I don't know<br>◯ I choose not to answer this question |
| Thinking about members of your family living in this household, what is your combined annual income, meaning the total pre-tax income from all sources earned in the past year? | ◯ $0 to $9,999<br>◯ $10,000 to $14,999<br>◯ $15,000 to $19,999<br>◯ $20,000 to $34,999<br>◯ $35,000 to $49,999<br>◯ $50,000 to $74,999<br>◯ $75,000 to $99,999<br>◯ $100,000 to $199,999<br>◯ $200,000 or more<br>◯ I choose not to answer this question |
| In the past year, have you or any family members you live with been unable to get any of the following when it was really needed? (Select all that apply)<br><br>If you HAVE been able to get all of the following, please select "I have been able to get all of the following" from the list of options. | ☐ Food<br>☐ Utilities<br>☐ Clothing<br>☐ Child care<br>☐ Medicine or Any Health Care (medical, dental, mental health, vision)<br>☐ Phone<br>☐ Other (please write): ______<br>☐ I have been able to get all of the following<br>☐ I choose not to answer this question |
| Has lack of transportation kept you from medical appointments, meetings, work, or from getting things needed for daily living? | ◯ No<br>◯ Yes (it has kept me from medical appointments)<br>◯ Yes (it has kept me from non-medical meetings, appointments, work, or from getting things I need)<br>◯ I choose not to answer this question |

| | |
|---|---|
| How often do you see or talk to people that you care about and feel close to?<br>(For example: talking to friends on the phone, visiting friends or family, going to church or club meetings) | ◯ Less than once a week<br>◯ 1 or 2 times a week<br>◯ 3 to 5 times a week<br>◯ 5 or more times a week<br>◯ I choose not to answer this question |
| Stress is when someone feels tense, nervous, anxious, or can't sleep at night because their mind is troubled. How stressed are you? | ◯ Not at all<br>◯ A little bit<br>◯ Somewhat<br>◯ Quite a bit<br>◯ Very much<br>◯ I choose not to answer this question |
| Are you a refugee? | ◯ No<br>◯ Yes<br>◯ I choose not to answer this question |
| Do you feel physically and emotionally safe where you currently live? | ◯ No<br>◯ Yes<br>◯ Unsure<br>◯ I choose not to answer this question |
| In the past year, have you been afraid of your partner or ex-partner? | ◯ No<br>◯ Yes<br>◯ Unsure<br>◯ I have not had a partner in the last year<br>◯ I choose not to answer this question |
