# Supplementary material for: Correlates of Fitness Tracker Ownership and Use in Cancer Survivors: Cross-Sectional Survey
Source: JMIR Cancer. 2026 May 26;12:e92876. doi: 10.2196/92876 (PMC13250496; doi:10.2196/92876)
Supplement: Multimedia Appendix 2 [file cancer_v12i1e92876_app2.docx]

| **Table S1.** Full multivariable logistic regression predicting fitness-tracker ownership with all candidate predictors entered simultaneously (n=869).^a^ | | |
| --- | --- | --- |
| **Variable** | **OR (95% CI)** | ***P*** |
| Age, per year increase | 0.99 (0.97–1.00) | .058 |
| **Sex** |  |  |
| Female | Ref. |  |
| Male | **0.61 (0.42–0.89)** | **.010** |
| **Race/Ethnicity** |  |  |
| White, non-Hispanic | Ref. |  |
| Other | 0.91 (0.51–1.63) | .760 |
| **Annual Household Income** |  |  |
| $0–$49,999 | Ref. |  |
| $50,000–$99,999 | 1.49 (0.97–2.31) | .071 |
| $100,000+ | **3.07 (1.95–4.83)** | **<.001** |
| No response | 1.36 (0.84–2.23) | .215 |
| **Education** |  |  |
| High school diploma/GED or less | Ref. |  |
| Some college | 0.85 (0.50–1.45) | .560 |
| College degree | 1.32 (0.81–2.15) | .261 |
| Graduate/professional degree | 1.08 (0.64–1.81) | .769 |
| **Cancer Site** |  |  |
| Breast | Ref. |  |
| Thyroid | **0.61 (0.39–0.97)** | **.035** |
| Oral cavity/pharynx | **0.39 (0.24–0.64)** | **<.001** |
| Bone marrow | **0.47 (0.25–0.89)** | **.020** |
| All other sites | **0.50 (0.32–0.77)** | **.002** |
| **Perceived Stress Level** |  |  |
| Not at all | Ref. |  |
| A little bit | 0.92 (0.58–1.46) | .709 |
| Somewhat | 1.12 (0.69–1.84) | .647 |
| Quite a bit/very much | 1.08 (0.62–1.89) | .784 |
| **Confidence in Self-Care** |  |  |
| Completely confident | Ref. |  |
| Very confident | 0.93 (0.57–1.50) | .762 |
| Somewhat confident | 0.84 (0.50–1.41) | .507 |
| A little/not at all confident | 0.95 (0.49–1.85) | .890 |
| **Physical Activity Frequency** |  |  |
| Every day | Ref. |  |
| 4–5 days/week | 0.81 (0.53–1.24) | .327 |
| 2–3 days/week | 0.85 (0.54–1.34) | .490 |
| <2 days/week | 0.67 (0.42–1.07) | .092 |
| **BMI Category, kg/m²** |  |  |
| <25.0 (Normal weight) | Ref. |  |
| 25.0–29.9 (Overweight) | 1.21 (0.82–1.78) | .344 |
| ≥30.0 (Obese) | 1.14 (0.77–1.71) | .509 |

**Abbreviations:** BMI, body mass index; CI, confidence interval; GED, General Educational Development; OR, odds ratio; Ref., reference category.

^a^ All candidate predictor variables were entered simultaneously into a single multivariable logistic regression model (n = 869). This sensitivity analysis was conducted to confirm that the direction and magnitude of associations were consistent with the reduced model presented in Table 2. Variables included in the model were mutually adjusted.

**Bold values indicate statistical significance at *P* < .05. *P* values are two-sided.**

| **Table S2.** Sensitivity analysis: full logistic regression model predicting frequent fitness-tracker use among current owners with all candidate predictors entered simultaneously (n = 504). | | |
| --- | --- | --- |
| **Variable** | **OR (95% CI)** | ***P*** |
| Age, per year increase | 1.00 (0.98–1.02) | .99 |
| **Sex** |  |  |
| Female | Ref. |  |
| Male | 0.99 (0.54–1.81) | .96 |
| **Reported Barriers to Use** |  |  |
| Accuracy concerns | **2.11 (1.03–4.30)** | **.040** |
| Discomfort (irritation, bulkiness) | **0.24 (0.12–0.48)** | **<.001** |
| Not tech savvy | 1.02 (0.46–2.22) | .97 |
| Battery life | 0.70 (0.41–1.21) | .20 |
| Information overload | 0.45 (0.20–1.02) | .055 |
| Low motivation | **0.21 (0.11–0.42)** | **<.001** |
| Privacy concerns | 0.70 (0.20–2.53) | .59 |
| Additional costs | 1.28 (0.43–3.84) | .66 |
| Limited app/device integration | **0.22 (0.08–0.63)** | **.004** |
| Device malfunction | **0.23 (0.09–0.60)** | **.003** |
| Limited internet access | 0.55 (0.12–2.58) | .45 |
| No one to help me use it | 2.47 (0.51–11.89) | .26 |
| Other | 0.35 (0.10–1.21) | .096 |

^a^ All candidate predictors were entered simultaneously into a single logistic regression model. The model included 504 device owners with complete data. Frequent use was defined as wearing the device every day or most days (n = 416); the reference category was infrequent use (rarely, some days, or never; n = 88).

**Bold values** indicate statistical significance at P < .05. P values are two-sided.

**Abbreviations:** CI, confidence interval; OR, odds ratio; Ref., reference category.

| **Table S3.** Sensitivity analysis: full logistic regression model predicting every-day fitness-tracker use among current owners with all candidate predictors entered simultaneously (n = 504). | | |
| --- | --- | --- |
| **Variable** | **OR (95% CI)** | ***P*** |
| Age, per year increase | 1.01 (1.00–1.03) | .16 |
| **Sex** |  |  |
| Female | Ref. |  |
| Male | 0.85 (0.51–1.41) | .52 |
| **Reported Barriers to Use** |  |  |
| Accuracy concerns | **1.80 (1.02–3.15)** | **.042** |
| Discomfort (irritation, bulkiness) | **0.18 (0.09–0.34)** | **<.001** |
| Not tech savvy | 0.77 (0.40–1.46) | .42 |
| Battery life | **0.60 (0.38–0.95)** | **.030** |
| Information overload | 0.74 (0.35–1.57) | .44 |
| Low motivation | **0.32 (0.17–0.60)** | **<.001** |
| Privacy concerns | 1.29 (0.37–4.51) | .69 |
| Additional costs | 1.80 (0.67–4.87) | .25 |
| Limited app/device integration | **0.30 (0.11–0.82)** | **.019** |
| Device malfunction | **0.32 (0.13–0.79)** | **.013** |
| Limited internet access | 1.14 (0.26–4.92) | .87 |
| No one to help me use it | 1.51 (0.44–5.21) | .51 |
| Other | 0.54 (0.17–1.68) | .29 |

^a^ All candidate predictors were entered simultaneously into a single logistic regression model. The model included 504 device owners with complete data. Every-day use was defined as wearing the device every day (n = 336); the reference category was all other use frequencies (most days, some days, rarely, or never; n = 168).

**Bold values** indicate statistical significance at P < .05. P values are two-sided.

**Abbreviations:** CI, confidence interval; OR, odds ratio; Ref., reference category.
